# Slope Entropy Characterisation: The Role of the *δ* Parameter

**DOI:** 10.3390/e24101456

**Published:** 2022-10-12

**Authors:** Mahdy Kouka, David Cuesta-Frau

**Affiliations:** 1 Department of System Informatics and Computers, Universitat Politècnica de València, 03801 Alcoy, Spain; 2 Technological Institute of Informatics, Universitat Politècnica de València, 03801 Alcoy, Spain

**Keywords:** slope entropy, time series classification, parameter optimisation, permutation entropy

## Abstract

Many time series entropy calculation methods have been proposed in the last few years. They are mainly used as numerical features for signal classification in any scientific field where data series are involved. We recently proposed a new method, Slope Entropy (SlpEn), based on the relative frequency of differences between consecutive samples of a time series, thresholded using two input parameters, γ and δ. In principle, δ was proposed to account for differences in the vicinity of the 0 region (namely, ties) and, therefore, was usually set at small values such as 0.001. However, there is no study that really quantifies the role of this parameter using this default or other configurations, despite the good SlpEn results so far. The present paper addresses this issue, removing δ from the SlpEn calculation to assess its real influence on classification performance, or optimising its value by means of a grid search in order to find out if other values beyond the 0.001 value provide significant time series classification accuracy gains. Although the inclusion of this parameter does improve classification accuracy according to experimental results, gains of 5% at most probably do not support the additional effort required. Therefore, SlpEn simplification could be seen as a real alternative.

## 1. Introduction

In recent years, entropy estimation methods have become very popular among scientists to extract hidden information from time series [1,2,3,4]. These methods basically compute the relative frequency of a set of numerical subsequences or symbolic patterns [5,6], and from this set, the entropy, complexity, predictability, or amount of information in the time series [7]. Many definitions apply, and it is estimated using a quantifier such as Shannon Entropy [8].

A lot of scientific fields have benefited from the high segmentation power of these methods [9,10], which have been used as features to distinguish among time series types in classification or clustering algorithms [11,12]. For example, in biomedicine, probably the field where these methods have been more extensively used so far, they have been used in almost all medical disciplines: to detect pathological states from electroencephalograms [13], from electrocardiograms-RR time series [14], from body temperature time series [15], and from actigraphy records [16], among many others. In industry, entropy methods have been used for predictive machinery maintenance using vibration time series [17]. In the economy, some of these methods have been used for financial time series analysis [18]. There are other applications in many fields, such as seismography [19], ecology [20], climatology [21], and many more.

Each one of the current entropy calculation methods available nowadays obviously has its strengths and weaknesses, and not all of them are suitable for any time series. Method capabilities and time series properties matching must be optimised in advance. For example, Approximate Entropy (ApEn) [1] is very sensitive to the input time series number of samples [22], Permutation Entropy (PE) [23] does not take into account time series amplitude in its calculations [3], and Sample Entropy (SampEn) [24] is very dependent on the sampling frequency [25]. In general, all the methods are very dependent on the values of their input parameters [22].

Another time series feature that might affect entropy computation, specifically in symbolic methods, such as PE, is the presence of contiguous equal samples or ties [26]. If two consecutive values are equal, they will be located one next to the other once their data subsequence is sorted, and the resulting ordinal pattern to be assigned will be impossible to uniquely determine. As a consequence, the relative frequency of symbolic patterns can be altered depending on the final sorting rule adopted to eliminate this ambiguity, and, therefore, the PE result of the time series will certainly vary. Although the influence of these variable results on signal classification is not high and has already been characterised [27], it could lead to misinterpretations about other time series properties [28].

A recently proposed method, Slope Entropy (SlpEn) [29], specifically included a symbolic interval around the 0 value for the difference between consecutive samples (null gradient) and explicitly accounts for the possible ties. In this case, there is no ambiguity, and the results remain unchanged since no additional rule has to be chosen. This interval is controlled by the δ input parameter of SlopEn. Instead of trying to avoid this uniformity of values in the same subsequence as in PE, the δ parameter transforms equalities into symbol 0; namely, they are an intrinsic part of the calculations.

However, this SlpEn feature has not been characterised yet. In other words, Is it really necessary to account for ties in SlpEn since in other methods, such as PE as stated above, it was demonstrated not to be significant from a time series classification perspective? SlpEn has been used in several classification studies with very good results [29], but the effect of this interval in the vicinity of 0-difference remains to be really quantified. Moreover, instead of using this δ parameter for the 0 region only, it could be configured more freely to maximise classification performance, if possible, in coordination with the other SlpEn parameter γ. Last but not least, if the influence of the δ parameter was minor or negligible, the SlpEn method could be further simplified.

The characterisation of δ role in SlpEn is addressed in the present work, including the expansion of this interval beyond such 0-difference and the classification accuracy improvement it really entails. Our study will quantify the influence and possible additional roles of this specific interval and parameter (δ) within a general context of SlpEn characterisation. It will be necessary to find out numerically whether it has a significant impact on the classification performance achievable or exhibits some kind of redundancy or diminishing return. In order to reach that goal, two variations of the standard SlpEn method will be applied to a heterogeneous set of time series: the complete SlpEn method and the SlpEn method without the 0-interval (redistributed between the two contiguous intervals since δ will be excluded). In all cases, the input parameters, along with $\delta =1\times 10^{-3}$ (if in use), will be varied to find the optimal combination and maximise the classification performance of the experimental datasets. The time series analysed include ties in their original form, essential to assess their possible correlation with the 0-interval symbol. The classification accuracy will be compared in terms of the percentage of correctly classified time series, with or without δ. Finally, the δ parameter will be freely varied for SlpEn computation to assess whether δ can have a similar role to γ or not beyond its usual $1\times 10^{-3}$ value.

The structure of the paper is as follows. In the next section, the SlpEn method, and for comparative purposes, the PE method (another method that could be influenced by ties), will be described in detail. In this section too, the type of time series included in the experimental datasets will be outlined. In Section 3, the experiments conducted will be reported, as well as the results achieved. After this section, a thorough discussion of these results will be presented. The last section of the paper will be devoted to the conclusions.

## 2. Materials and Methods

### 2.1. Datasets

The experimental dataset comprises several types of time series with different features in terms of bandwidth, length, ties and regularity. All the records used in this study are publicly available, and many of them have already been used in similar studies. The specific time series employed in the experiments were drawn from the databases:

- The Bern–Barcelona database [30]. This database contains focal and non-focal time series from seizure-free records. We used 50 records of length 10,240 samples from each class, sampled at 512 Hz.
- The Fantasia database [31]. A total of 40 records from this database were used in this study: 20 records from old subjects and 20 from young subjects. They were healthy subjects that were monitored for 120 min. The specific signals used were the RR intervals.
- The Ford A dataset [32]. These time series were collected from an automotive subsystem. The goal was to assess if a classification scheme could distinguish those cases where a certain symptom existed in a subsystem based on engine noise. We used 40 records from each class.
- The House Twenty dataset [33,34,35]. Time series collected as part of the project Personalised Retrofit Decision Support Tools for UK Homes using Smart Home Technology (REFIT). This dataset includes data from 20 households. It is composed of two classes. The first class is the overall household electricity consumption. The second class is the overall dryer and washing machine electrical consumption.
- The PAF prediction dataset [36]. This database was created to assess the performance of algorithms for predicting Paroxysmal Atrial Fibrilation (PAF). We have used the 5-min records that correspond to patients that have PAF, considering two classes: records immediately preceding the PAF episode and records from a period distant from any PAF episode, the 25 first files in each case.
- The Worms two-class dataset [37,38]. This is a database that contains time series related to the movement of a specific type of worm to study behavioural genetics. We have used records from the two class problems of mutant vs. non-mutant worms. We extracted 75 records of 900 samples from type 1 and 105 from type 2.
- The Bonn EEG dataset [39,40]. This is one of the free epilepsy datasets available. It contains records of 4097 samples corresponding to 23.6 s from 5 classes: healthy subjects with eyes open (A), healthy subjects with eyes closed (B), and epileptic subjects (C, D, and E), with data recorded at the hemisphere opposite to the epileptogenic zone, seizure-free periods at the epileptogenic zone, and seizure activity from the hippocampal focus, respectively. There are 100 single-channel records from each class. We used the experimental records from classes D and E only.

### 2.2. Permutation Entropy Method

PE [23] is another method that can be influenced by the presence of ties in the input subsequences extracted from time series [28,41], but with very good performance in classification applications [42,43]. This method has been included in the study for reference and comparative purposes with the SlpEn method under analysis.

PE considers all the consecutive and overlapping subsequences of length *m* present in a time series x of length *N*. For each subsequence commencing at sample *j* and of length *m*, $x_{j}^{m}$, a sorting procedure in ascending (or descending) order of the samples of $x_{j}^{m}$ takes place.

As a result, from the initial indices of the samples in $x_{j}^{m}$ in their default order, {0,1,…,m−1}, a new symbolic pattern emerges with the final index of each sample once sorted at its corresponding location. This symbolic vector is represented as $\pi_{j}^{m}=\left\{\pi_{0},\pi_{1},\ldots ,\pi_{m-1}\right\}$ such that $\pi_{0}$ is the initial index of the smallest sample in $x_{j}^{m}$, $\pi_{1}$ the index of the next sample of $x_{j}^{m}$ in ascending order, and so on. In other words, the samples in $x_{j}^{m}$ satisfy $x_{j+\pi_{0}}\leq x_{j+\pi_{1}}\leq x_{j+\pi_{2}}\leq \ldots \leq x_{j+\pi_{m-1}}$.

All the possible m! ordinal patterns emerging from the input time series x are accounted for in a histogram, from which their relative frequencies $p_{j}$ are computed and used to obtain their Shannon entropy, which corresponds in this specific case to their PE:

(1) $$\begin{array}{c} \mathrm{PE}\left(x,m\right)=-\sum_{k=0}^{m!-1}p_{k}\log p_{k},\forall p_{k}>0. \end{array}$$

### 2.3. Slope Entropy Method

SlpEn [29,44] is also a symbolic entropy calculation method. For each subsequent $x_{j}^{m}$ in a time series x of length *m*, it computes the difference between consecutive samples, $x_{i}-x_{i-1}$, and assigns a symbol to that difference according to the input value of two parameters, γ, and δ (γ>δ), and the following rules:

- If $x_{i}>x_{i-1}+\gamma$, the maximum difference considered, the symbol assigned is +2 (or any other alphanumeric symbol from any alphabet).
- If $x_{i}>x_{i-1}+\delta$ and $x_{i}\leq x_{i-1}+\gamma$, the symbol assigned is +1.
- For the region supposedly close to a gradient or slope 0 (equal consecutive values or ties), when $|x_{i}-x_{i-1}|\leq \delta$ (with δ close to 0), the symbol assigned is 0. This symbol will be removed from the analysis to assess the real influence of δ on SlpEn, the main objective of the present study.
- If $x_{i}<x_{i-1}-\delta$ and $x_{i}\geq x_{i-1}-\gamma$, the symbol assigned is −1.
- Finally, if $x_{i}<x_{i-1}-\gamma$, the symbol assigned is −2.

Once all the symbolic patterns have been computed, and the number of matches for each case normalised by the number of unique patterns found [45,46], SlpEn is also calculated from the Shannon entropy of these approximated probabilities $p_{k}$:

(2) $$\begin{array}{c} \mathrm{SlpEn}\left(x,m,\gamma ,\delta \right)=-\sum_{\forall k}^{}p_{k}\log p_{k} \end{array}$$

**Figure 1:**
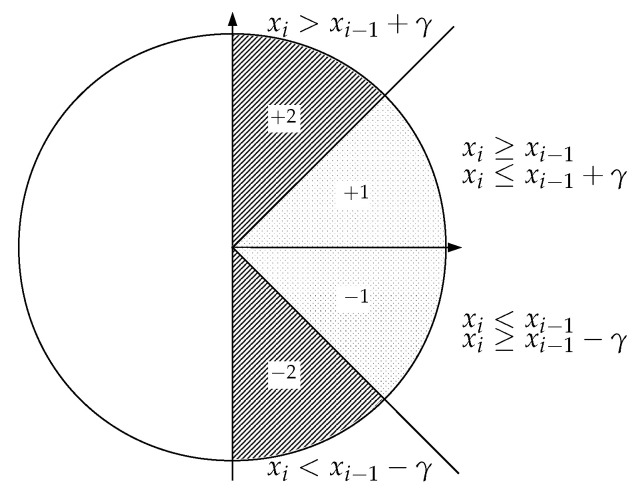
Graphical interpretation of the SlopEn approach removing the δ parameter. Possible symbols are now +2, +1, −1, and −2, depending on the amplitude difference between consecutive samples.

The role of the centre interval around gradient 0 and that of the δ parameter is what is studied in this paper. This parameter has usually been assigned a default fixed value of $1\times 10^{-3}$ to account for possible ties [16,47], but no other possibilities have been explored so far. If the time series classification accuracy (percentage of correctly classified time series over the total dataset) achieved using a SlpEn version without that interval was comparable to the accuracy of the complete method, the SlpEn method could be safely simplified without a detrimental impact on its distinguishing power. On the contrary, if other δ parameter setups offered a significant classification improvement, it would become advisable to vary δ as well as γ for performance maximisation. The effect of removing the δ parameter from the SlpEn computation is depicted in Figure 1.

## 3. Experiments and Results

### 3.1. Experiments

The experiments were devised to assess the influence of δ on SlpEn and find out whether the symbolic interval for slopes around 0 was really necessary or not or if the classification accuracy could be improved. In order to accomplish that purpose, two versions of SlpEn were used in the experiments: the original version, termed SlpEn${}^{I}$, including δ, and a modified version, termed SlpEn${}^{II}$, where the interval based on δ was simply removed, and the differences between consecutive samples were assigned to neighbouring intervals +1 and −1.

The values of the input parameters were chosen using a grid search to find the optimal configuration in terms of the highest classification accuracy using the experimental datasets. The *m* parameter ranged from 3 up to 9, and the γ parameter from 0.10 up to 0.90 in 0.05 steps. The common parameters for both versions of SlpEn were *m* and γ, and only *m* for PE. The third SlpEn parameter, δ, was set to 0.001 as in the original method [29] and other works [16,47], and varied for classification analysis, with 0.10≤δ<γ. It was not used in the SlpEn version where the 0 interval was not considered, SlpEn${}^{II}$. In other words, the simplified version of SlpEn was where δ=0. Input records were normalised for a 0 average and unit standard deviation. Classification was based on a single threshold obtained from the ROC curve [48,49], computing the percentage of correctly classified time series in the dataset according to such threshold as the accuracy metric.

#### 3.1.1. Equal Values in Time Series and δ

A grid search took place to find the input *m* and γ parameter values that maximised the classification accuracy for the two classes in each dataset, with $\delta =1\times 10^{-3}$ as usual. As stated above, SlpEn${}^{I}$ refers to the standard method, including the 0 interval with δ constant, and SlpEn${}^{II}$ to the modified version without δ.

These results are reported in Table 1 and Table 2, including the optimal configuration found of the input parameters, the percentage of ties present originally in the data (considering a tie when two consecutive samples are equal), and the classification accuracy achieved for each dataset using the three methods compared.

#### 3.1.2. Parameter δ Optimisation

In this case, the classification of the datasets entailed the inclusion of the δ parameter in the grid search as an additional parameter such as γ. Instead of using δ=0.001 to supposedly account for the possible ties, this parameter was varied between 0.10 and γ, 0.10≤δ<γ, in order to find out if the classification accuracy could be substantially increased. The results are shown in Table 3 and Table 4.

## 4. Discussion

In the definition of the original SlpEn method [29], the 0 symbol was proposed to account for differences in the 0 region, and this has been later applied using δ=0.001 as the most frequent value used [15,47]. However, this scheme had not been characterised yet, and the possible benefit of using a specific symbol for the region of ties had to be assessed, the main objective of the present study.

The analysis of the influence of the standard δ=0.001 was reported in Table 1 and Table 2 using all the experimental datasets. In general, the complete method SlpEn${}^{I}$ yielded higher classification accuracy than the simplified version. The difference was highest for the Bern dataset, 80% against 76%, with a 3% difference for the Fantasia database, and negligible differences, if any, for the rest of datasets except the PAF database. In this case, the simplified method, SlpEn${}^{II}$, yielded the highest accuracy, 80% against 76%, despite significant differences in ties between the two classes under analysis, 6.72±8.81 and 19.68±11.13. This case is similar to that of the House database, with different levels of ties, 27.58±11.67 and 23.31±10.47, but no differences in classification accuracy.

On the contrary, there are cases without significant differences in the percentage of ties, such as in the Bern and FordA databases, but the complete method achieved a higher classification accuracy. This lack of a clear correlation between ties and performance with δ suggests that this parameter is looking at more differences between classes than just the ties, but, from a classification accuracy point of view, its role is probably minor and unnecessary. In fact, computing averages of all the classification results just for comparative purposes, the difference is between 83.28±9.08 for SlpEn${}^{I}$ and 82.42±9.10 for SlpEn${}^{II}$ which is clearly not significant. Therefore, these results suggest the original SlpEn method could be further simplified without δ, saving computing time and memory requirements for the algorithm but without any significant detrimental impact on classification accuracy. Only for very difficult classification problems with low accuracy, would the complete method be advisable.

The other entropy calculation method tested, PE, yielded results below those of SlpEn${}^{I}$ and SlpEn${}^{II}$. The difference was quite great for databases Bern, Fantasia, Ford, and House, with an almost 20% difference in most cases. For Worms and Bonn, the results were quite similar, and only in the PAF case PE outperformed SlpEn${}^{I}$ and SlpEn${}^{II}$. PE is a method extensively studied and successfully applied for classification purposes due to its simplicity and robustness, and these results confirm once more the goodness of SlpEn${}^{I}$. This also applies to SlpEn${}^{II}$, and, therefore, supports the simplification of the standard SlpEn method since the classification accuracy is still better than that of such a good method as PE.

Regarding the results of δ parameter optimisation reported in Table 3 and Table 4, there is also a minor improvement in the classification accuracy. Bern goes from 80% to 81%, Fantasia from 85% to 87%, FordA stays at 83%, House from 95% to 100%, PAF from 76% to 84%, the greatest improvement, and Bonn from 94% to 95%. The only case where the accuracy worsens is the Worms dataset, from 70% to 67%, but this is due to the different δ values, originally δ=0.001, and in this search, it started at δ=0.10.

Considering the maximum improvement achieved using this full customisation of δ, it is still not clear if the additional computational burden is worth the effort. The grid search of SlpEn${}^{II}$ computational complexity is quadratic (*m* and γ optimisation), but with full optimisation, it becomes cubic (m,γ,δ optimisation). The simplified method still outperforms most of the entropy methods available, and it is probably the best option, especially in resource-constrained computational systems, such as embedded systems (medical or IoT devices, among others). For comparative purposes, Table 5 shows the processing time of each dataset using SlpEn${}^{I}$ and SlpEn${}^{II}$ on a PC in seconds.

## 5. Conclusions

This paper assessed the role of the δ parameter for time series classification using SlpEn. It was originally devised to account for possible ties in the data, but in this case, it was also customised as an additional parameter, such as γ, in order to quantify its influence on classification accuracy.

In both cases, this study demonstrated that the improvement achieved using the additional parameter δ is not worth the effort, falling within the scope of the law of diminishing returns. Without that parameter, the simplified SlpEn method still achieved a high classification accuracy, comparable to or even higher than that achieved with a similar and widely used entropy method such as PE. Removing that parameter, the comparison for the 0 symbol is no longer necessary, and the grid search for parameter optimisation can only focus on *m* and γ, saving memory and, mainly, computational load.

In general, if classification accuracy is not critical, it is reasonable to use the simplified version of SlpEn without the δ parameter. If the time series under study are difficult to classify, the computational resources are almost limitless, or if the results are borderline in terms of statistical significance, then those marginal returns provided by δ could be exploited.

Since marginal returns are still returns, further studies should focus on optimising the computational cost of SlpEn in order to make the addition of the δ parameter more efficient. SlpEn is already a very simple algorithm, with a single iteration through the data, in contrast to ApEn or SampEn, and no need for sorting as in PE. However, the addition of another parameter optimised with a grid search entails increasing the computational cost exponentially. With parameters *m* and γ only, SlpEn falls in the realm of ApEn or SampEn optimisation but at a clear disadvantage against PE. Even the addition of more parameters/thresholds and the application of a non-symmetrical scheme (not the same threshold values in the positive and negative regions of Figure 1) should be studied to assess the possibility of extra classification accuracy improvements.

## Figures and Tables

**Table 1:** Optimal input parameter configuration and results obtained using the experimental dataset in its original state in terms of ties. Experimental datasets: Bern–Barcelona, Fantasia, Ford A, and House Twenty.

		Experimental Dataset			
		Bern–Barcelona	Fantasia	FordA	House Twenty
Baseline	Class 1	0.00±0.00	8.05±4.40	0.00±0.00	27.58±11.67
ties (%)	Class 2	0.00±0.00	3.61±1.80	0.48±0.29	23.31±10.47
Baseline	SlpEn${}^{I}$	80%(m=6,γ=0.85)	85%(m=5,γ=0.5)	83%(m=3,γ=0.4)	95%(m=3,γ=0.1)
classification	SlpEn${}^{II}$	76%(m=7,γ=0.15)	82%(m=4,γ=0.1)	82%(m=9,γ=0.3)	95%(m=3,γ=0.1)
results	PE	60%(m=9)	65%(m=4)	75%(m=6)	67%(m=9)

**Table 2:** Optimal input parameter configuration and results obtained using the experimental dataset in its original state in terms of ties. Experimental datasets: Worms, PAF, and Bonn.

		Experimental Dataset		
		Worms	PAF	Bonn
Baseline	Class 1	4.40±2.81	6.72±8.81	4.72±1.76
ties (%)	Class 2	6.06±7.83	19.68±11.13	1.12±0.95
Baseline	SlpEn${}^{I}$	70%(m=5,γ=0.15)	76%(m=4,γ=0.85)	94%(m=4,γ=0.10)
classification	SlpEn${}^{II}$	69%(m=3,γ=0.55)	80%(m=4,γ=0.60)	93%(m=9,γ=0.10)
results	PE	68%(m=7)	82%(m=3)	91%(m=3)

**Table 3:** Optimal input parameter configuration, including δ, and results obtained using the experimental datasets Bern, Fantasia, FordA, and House.

	Experimental Dataset			
	Bern–Barcelona	Fantasia	FordA	House Twenty
SlpEn${}^{I}$	81%	87%	83%	100%
	(m=8,γ=0.20,δ=0.15)	(m=8,γ=0.55,δ=0.50)	(m=7,γ=0.40,δ=0.15)	(m=3,γ=0.15,δ=0.05)

**Table 4:** Optimal input parameter configuration, including δ, and results obtained using the experimental datasets Worms, PAF, and Bonn.

	Experimental Dataset		
	Worms	PAF	Bonn
SlpEn${}^{I}$	67%	84%	95%
	(m=9,γ=0.20,δ=0.05)	(m=3,γ=0.75,δ=0.65)	(m=8,γ=0.10,δ=0.05)

**Table 5:** Computation time(s) for each experimental dataset using both variants of SlpEn.

	SlpEn${}^{I}$	SlpEn${}^{II}$
Bonn	22.50 s	18.52 s
Fantasia	4.89 s	3.45 s
Bern	34.70 s	29.77 s
FordA	0.28 s	0.22 s
House	0.26 s	0.22 s
PAF	0.60 s	0.55 s
Worms	1.70 s	1.40 s

## Data Availability

Not applicable.
